# Immune Modulation in Prostate Cancer Patients Treated with Androgen Receptor (AR)-Targeted Therapy

**DOI:** 10.3390/jcm9061950

**Published:** 2020-06-22

**Authors:** Vincenza Conteduca, Orazio Caffo, Emanuela Scarpi, Pierangela Sepe, Luca Galli, Lucia Fratino, Francesca Maines, Vincenzo Emanuele Chiuri, Matteo Santoni, Elisa Zanardi, Francesco Massari, Ilaria Toma, Cristian Lolli, Giuseppe Schepisi, Andrea Sbrana, Stefania Kinspergher, Maria Concetta Cursano, Chiara Casadei, Caterina Modonesi, Daniele Santini, Giuseppe Procopio, Ugo De Giorgi

**Affiliations:** 1 Department of Medical Oncology, Istituto Scientifico Romagnolo per lo Studio e la Cura dei Tumori (IRST) IRCCS, Via Piero Maroncelli 40, 47014 Meldola, Italy; emanuela.scarpi@irst.emr.it (E.S.); cristian.lolli@irst.emr.it (C.L.); giuseppe.schepisi@irst.emr.it (G.S.); tcursano@gmail.com (M.C.C.); chiara.casadei@irst.emr.it (C.C.); ugo.degiorgi@irst.emr.it (U.D.G.); 2 Department of Oncology, Ospedale Santa Chiara, 38122 Trento, Italy; orazio.caffo@apss.tn.it (O.C.); francesca.Maines@apss.tn.it (F.M.); Stefania.Kinspergher@apss.tn.it (S.K.); 3 Medical Oncology Department, Fondazione Istituto Nazionale dei Tumori, 20133 Milano, Italy; pierangela.sepe@istitutotumori.mi.it (P.S.); Giuseppe.procopio@istitutotumori.mi.it (G.P.); 4 Medical Oncology Unit 2, Polo Oncologico, Azienda Ospedaliero-Universitaria Pisana, 56126 Pisa, Italy; lugal71@yahoo.it (L.G.); andreasbrana89@gmail.com (A.S.); 5 Department of Medical Oncology, Centro di Riferimento Oncologico di Aviano, 33081 Aviano-Pordenone, Italy; lfratino@cro.it; 6 Department of Oncology, Ospedale Vito Fazzi, 73100 Lecce, Italy; chiuriv@yahoo.it; 7 Oncology Unit, Macerata Hospital, 62100 Macerata, Italy; mattymo@alice.it; 8 Academic Unit of Medical Oncology, IRCCS San Martino Polyclinic Hospital, 16132 Genoa, Italy; zanardielisa@yahoo.it; 9 Department of Internal Medicine and Medical Specialties (DIMI), School of Medicine, University of Genoa, 16132 Genoa, Italy; 10 Division of Oncology, S.Orsola-Malpighi Hospital, 40138 Bologna, Italy; francesco.massari@aosp.bo.it; 11 Clinical Oncology, Arcispedale Sant’Anna University Hospital, 44124 Ferrara, Italy; ilaria.toma88@gmail.com; 12 Department of Medical Oncology, Campus Bio-Medico University of Rome, 00128 Rome, Italy; d.santini@unicampus.it; 13 Ospedali Riuniti Padova SUD, 35043 Padua, Italy; caterina.modonesi@aulss6.veneto.it

**Keywords:** prostate cancer, androgen deprivation therapy, abiraterone, enzalutamide, autoimmunity, prognosis

## Abstract

Androgen deprivation therapy (ADT) is a cornerstone of treatment for prostate cancer and, in recent years, androgen receptor (AR)-targeted therapies (abiraterone and enzalutamide) have both been used for the treatment of castration-resistant prostate cancer (CRPC). In our study, we sought to investigate the association between ADT and immune disorders, considering a potential role of androgens in the immune modulation. We retrospectively evaluated CRPC patients treated with abiraterone/enzalutamide between July 2011 and December 2018. We assessed the risk of developing immune alterations and their impact on outcome. We included 844 CRPC patients receiving AR-directed therapies, of whom 36 (4.3%) had autoimmune diseases and 47 (5.6%) second tumors as comorbidities. Median age was 70 years [interquartile range (IQR) = 63–75)]. We showed higher significant incidence of autoimmune diseases during their hormone sensitive status (*p* = 0.021) and the presence of autoimmune comorbidities before starting treatment with abiraterone/enzalutamide was significantly associated with worse overall survival (OS) (10.1 vs. 13.7 months, HR = 1.59, 95% CI 1.03–2.27, *p* = 0.038). In a multivariate analysis, the presence of autoimmune disorders was an independent predictor of OS (HR = 1.65, 95% CI 1.05–2.60, *p* = 0.031). In conclusion, CRPC patients with autoimmune alterations before starting AR-directed therapies may have worse prognosis. Further prospective studies are warranted to assess the role of immune modulation in the management of prostate cancer patients.

## 1. Introduction

Prostate cancer is one of the most prevalent cancers worldwide and a frequent cause of cancer-related death in Western countries [1]. Androgen deprivation therapy (ADT) represents the cornerstone of treatment for prostate tumors, even though up to 20% of patients who undergo ADT will develop castration-resistant prostate cancer (CRPC) [2]. In this setting, potent androgen receptor (AR)-directed therapies (abiraterone and enzalutamide) [3,4,5,6] have an important role in the therapeutic algorithm of advanced prostate cancer.

However, despite being highly effective, hormonal treatments are associated with side effects that could negatively affect the patient’s quality of life and, in some cases, clinical outcome, such as musculoskeletal, metabolic and cardiovascular side effects [7,8,9]. Metabolic syndrome is a cluster of metabolic and cardiovascular risk factors including obesity, insulin resistance, diabetes, dyslipidemia and hypertension leading to cardiovascular diseases. Chronic low-grade inflammation has been also established as an important risk factor of metabolic syndrome [7]. Several cytokines, which play an important physiological role in different metabolic pathways, are also involved in the regulation of autoimmune and/or inflammatory processes. Alterations in the immune-metabolic crosstalk may also contribute to the development of autoimmune diseases [10,11]. 

The potential direct action of androgens in the immune modulation has been previously investigated [12]. Nevertheless, currently, limited evidences are available on the association between ADT and the occurrence of immune disorders.

Immunological alterations include a heterogeneous group of diseases spanning from autoimmune disorders to malignant diseases, in the context of escape mechanisms from immune tolerance and surveillance.

**Figure 1:**
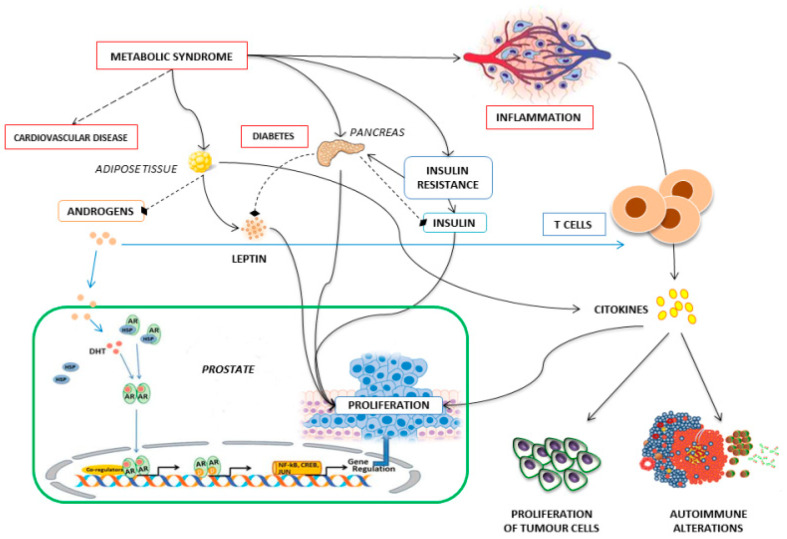
Alterations in the immune-metabolic crosstalk contribute to the development of autoimmune diseases. A relationship among the metabolic syndrome, inflammation and immune system leads to the secretion of cytokines that, in turn, may lead to an uncontrolled cell proliferation and autoimmune alterations.

Beyond its primary antitumor effect inducing apoptosis of tumor cells, ADT has been also indirectly lead to the priming of tumor-specific adaptive immune responses [13], impairing immune cell infiltrating, especially a T-cell subset, and the production of several inflammatory cytokines involved in the pathogenesis of numerous autoimmune diseases and in the regulation of tumor cell proliferation [14] (Figure 1). 

To date, the role of androgen in modulating immune function and the consequence of androgen removal on adaptive immune responses has been particularly investigated in in vivo and in vitro studies, considering androgen as an immunosuppressive factor and revealing a possible relationship between T-cell function and castration state in autoimmune disease models [15,16,17]. 

Our work represents the first clinical study aimed to investigate the incidence of immunological alterations (autoimmune disorders and, in an exploratory approach, the risk of developing second tumors) in advanced prostate cancer patients treated with abiraterone or enzalutamide.

## 2. Patients and Methods

### 2.1. Patient Population

This was a multi-institution study with the primary aim of evaluating the clinical impact of immune disorders in CRPC patients treated with abiraterone or enzalutamide. The secondary objective was to determine the correlation between the immunological alterations and ADT duration during the natural history of prostate tumor. Patients with metastatic CRPC treated with abiraterone or enzalutamide at 12 Italian institutions were retrospectively identified. All patients had histology of prostate adenocarcinoma without neuroendocrine differentiation. 

Therapy consisted of abiraterone 1000 mg daily associated with prednisone 5 mg twice daily or enzalutamide 160 mg daily. Treatment was given continuously until there was evidence of either progressive disease (PD) or unacceptable toxicity. Prior treatments for hormone -sensitive prostate cancer (HSPC) and CRPC included hormonal therapy and/or chemotherapy and/or radiometabolic therapy. The choice of each therapy was at the discretion of the treating physician. Patients with diagnosis of autoimmune diseases before starting ADT for prostate cancer were excluded.

The accuracy of all of the clinical, pathologic and radiologic data retrieved from the respective institutions’ databases was validated for each patient by an independent observer using the medical chart.

Radiographic progression was defined using Response Evaluation Criteria in Solid Tumours version 1.1. PSA decline was evaluated according to the Prostate Cancer Working Group (PCWG3) guidelines [18].

The study was conducted in accordance with the Declaration of Helsinki and the Good Clinical Practice guidelines. The protocol was approved by the independent review board at each participating site and written informed consent was obtained from patients. 

### 2.2. Diagnostic Criteria of Autoimmune Disease

We identified diagnoses of systemic autoimmune diseases and organ-specific autoimmune diseases (i.e., thyroiditis, Basedow disease, type I diabetes), rheumatic diseases (i.e., rheumatoid arthritis, psoriatic arthritis, etc.), systemic autoimmune diseases (i.e., Sjogren syndrome, sclerotic cholangitis, autoimmune hepatitis, etc.), neurologic-autoimmune-like diseases (i.e., myasthenia gravis, Guillain-Barré syndrome) and vasculitis. We defined and categorized autoimmune diseases according to the International Classification of Diseases, 10th Revision (ICD-10) codes (Table S1).

### 2.3. Statistical Analysis

Progression-free survival (PFS) was defined as the time from the start of treatment with abiraterone or enzalutamide until disease progression or death from any cause or last tumor evaluation. 

Overall survival (OS) was defined as the time from the start of AR-directed therapies until death from any cause or last follow-up. Survival curves were estimated by the Kaplan-Meier method. The log-rank test was performed to compare survival curves between groups of patients by the presence of immune alterations. A logistic regression model was used to investigate potential predictors of immune alterations and to evaluate the odds ratio (OR) and their 95% confidence intervals (95% CI). The median test (Wilcoxon) was performed to compare the median duration of ADT timing between patients with or without immune alterations. Chi-Square or Fisher’s exact test were performed to evaluate the association between the presence of concurrent autoimmune disease and PSA response, as appropriate.

All statistical analyses were carried out using SAS Statistical software version 9.4 (SAS Institute, Cary, NC, USA). For all the analysis, a two-sided *p*-value < 0.05 was considered as statistically significant.

## 3. Results

### 3.1. Patient and Treatment Characteristics

**Figure 2:**
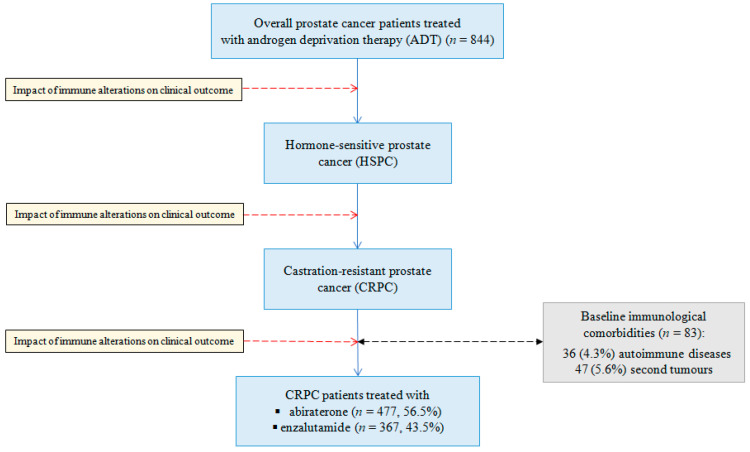
Flow chart of prostate cancer patient cohort included in this study.

We identified 844 patients with metastatic prostate cancer with a median age of 70 years (interquartile range (IQR) = 63–75)), between July 2011 and December 2018 (Figure 2). We observed Eastern Cooperative Oncology Group (ECOG) performance status 0–1 in 788 (93.4%) patients, and presence of visceral metastasis in 92 (10.9%) cases.

In our cohort, 335 out of 844 (39.7%) patients had de novo metastatic prostate cancer, the remaining patients developed a metastatic disease in a median time of 4.2 years (range: 2–17.3 years), receiving a prior radical therapy (radiation and/or prostatectomy) in most cases.

All patients received ADT for the HSPC stage, but the exact start date of ADT was missing in 61 (7.2%) cases. Median duration of HSPC was 29 months (IQR 14–59), whereas median CRPC duration was 22 months (IQR = 13–39). Most patients (*n* = 764, 90.5%) received ≤2 therapeutic lines for CRPC before starting abiraterone or enzalutamide. 

All patients underwent AR-directed therapy with abiraterone (*n* = 477, 56.5%) or enzalutamide (*n* = 367, 43.5%), of whom 189 (39.6%) and 170 (46.3%) were chemotherapy-naive, respectively. The number of therapeutic lines before treatment with abiraterone or enzalutamide was ≤2 in 90.5% of cases.

Clinical and treatment features of these patients are summarized in Table 1. 

### 3.2. Emergence of Autoimmune Disorders Before Starting AR-Directed Therapies 

Before treatment with abiraterone or enzalutamide, we reported comorbidities in 567 (67.2%) of 844 patients, particularly cardiovascular and metabolic alterations (Table 2).

Autoimmune disorders were reported in 36 (4.3%) of cases, of which arthritis (rheumatoid or psoriatic) and autoimmune thyroiditis were the most frequent (13 (1.5%) and 12 (1.4%), respectively). No difference in the incidence of immune disorders was reported between abiraterone- and enzalutamide-treated patients, and no correlation was observed between the incidence of cardiovascular and/or metabolic comorbidity and the presence of autoimmune disease.

In general, the incidence of autoimmune alterations before starting second-generation AR-directed therapies was significantly higher in their HSPC phase rather than CRPC stage (61% versus 39%, *p* = 0.021), we even observed a decreased time of HSPC status in men developing autoimmune disease compared to patients with no autoimmune alteration [19 months (range 1–100) vs. 30 (1–236) months, *p* = 0.044]. Conversely, no significant association was found with median duration of CRPC between patients with and without immune disorders (*p* = 0.354).

### 3.3. Autoimmune Comorbidities and Clinical Outcome to Abiraterone and Enzalutamide

**Figure 3:**
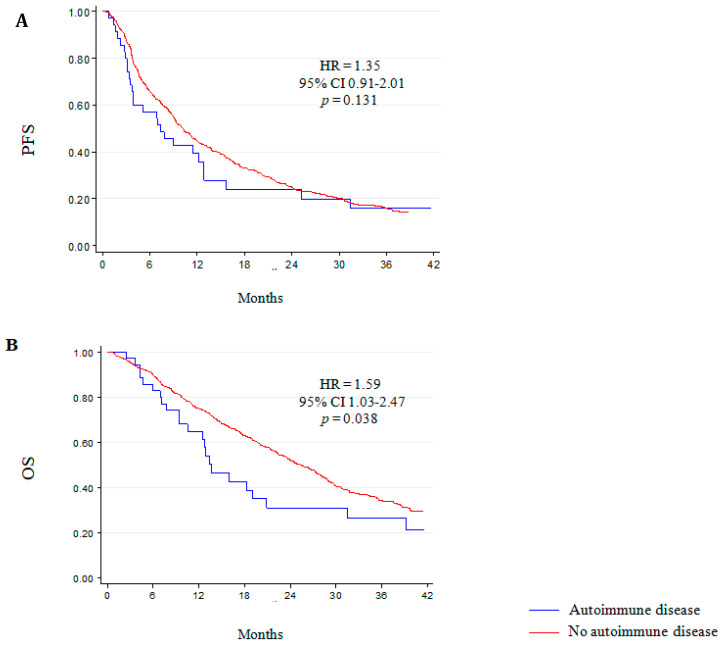
Clinical outcome and autoimmunity in castration-resistant prostate cancer patients treated with abiraterone or enzalutamide. Progression-free survival (PFS) (**A**) and overall survival (OS) (**B**) in patients with and without autoimmune disorders.

Median follow-up of our cohort was of 32 months (range 1–96), with a median PFS of 7.4 months (95% CI 3.7–12.8), and median OS of 13.7 months (95% CI 9.4–20.9). We observed no significant impact of the presence of concurrent autoimmune disease on PFS (Figure 3A) and PSA response (Table S2), and we reported a significant shorter median OS in patients with autoimmune disease compared to those without any autoimmune manifestation (10.1 months vs. 13.7 months, HR = 1.59, 95% CI 1.03–2.27, *p* = 0.038) (Figure 3B, Table S3). 

In our prespecified multivariate analysis including age, presence of autoimmune comorbidities, visceral metastasis, ECOG performance status, baseline PSA value and chemotherapy status, presence of cardiovascular and/or metabolic comorbidities (univariate analysis included in Table S3), presence of autoimmune disease was independently associated with OS (HR = 1.63, 95% CI, 1.03–2.57) along with other included factors (age: HR = 1.015, 95% CI 1.015–1.029, *p* = 0.042; visceral metastasis HR = 1.82, 95% CI 1.35–2.45, *p* < 0.0001; ECOG: HR = 3.30, 95% CI 2.37–4.58, *p* < 0.0001; pretreatment log- PSA: HR = 1.41, 95% CI 1.32–1.49, *p* < 0.0001; previous chemotherapy: HR = 1.74, 95% CI 1.38–2.19, *p* < 0.0001) (Table 3).

During AR-directed treatment, we reported no immune toxicity related to potent AR-directed therapies, according to the NCI-CTCAE v.4 (Table S4). The most common adverse event (5.9%) was weakness. Thirty-nine patients (4.6%) developed a metabolic syndrome, of whom 9 (23%) had one prior immune manifestation.

### 3.4. Risk of Developing Second Tumors

In an exploratory manner, we evaluated the emergence of second tumors during the history of prostate malignancy as an imbalance of immune regulation. Specifically, we assessed an incidence of second tumors in 47 (5.6%) cases, whose 38 were solid tumors. More common sites of secondary malignancies were colon-rectum (*n* = 13, 1.5%) and bladder (*n* = 10, 1.2%). Only four patients developed both autoimmune manifestation and second tumor. We showed a trend towards a significant association between higher incidence of second tumors during HSPC rather than CRPC status (*p* = 0.06).

## 4. Discussion

In this large study, we first reported the absence of immune alterations associated with AR-directed treatment in CRPC patients, but a negative impact of baseline immune disorders was observed on overall survival.

We observed a direct relationship between the duration of the hormone-sensitive phase and increased risk of autoimmune diseases in prostate cancer patients. Specifically, we evidenced a higher incidence of autoimmune manifestations in localized prostate cancer or HSPC. Several possible mechanisms can explain the effects of ADT use, as a primary treatment of HSPC, on the etiology of autoimmune diseases. Chronic inflammation has previously been found to be associated with both prostate cancer and autoimmune diseases [19]. Indeed, inflammation status, including also the release of inflammatory cytokines such as interleukin (IL)-1, IL-6 and IL-17, has been associated with the development and progression of prostate cancer [7,12,19], and androgens have been reported to alter T-cell immunity [17,20,21]. Moreover, ADT has been revealed to reduce Th1 and Th17 responses and also the concentration of inflammatory cytokines involved in several autoimmune diseases, including IL-1β, IL-2, tumor necrosis factor (TNF)-α and interferon (IFN)-γ [17,19].

In the present study, the biggest impact of hormonal treatment in developing immune alterations has been mainly reported in HSPC. The lack of any autoimmune events related to AR-directed therapy administered for CRPC could be explained to by the crucial role of the induction of strong anti-tumor CD8+ T cell responses with a concomitant increase in CD4+CD25+FoxP3+ Tregs following castration [17], which are mainly involved in the suppression of autoimmune manifestations. 

The association between ADT duration and immunological disorders has been also reported in an Asian population-based nationwide cohort study of 17,168 patients newly diagnosed with prostate cancer [22], resulting in a significant decreased risk of autoimmune diseases with longer ADT duration. In comparison, recently, Yang et al. [23] reported a 23% increased risk of rheumatoid arthritis in 44,785 patients who received ADT for prostate tumor in North America, arguing that the androgen-mediated thymic regeneration could be responsible for higher risk of rheumatoid arthritis, and reporting an increased risk of being diagnosed with rheumatoid arthritis with a longer duration of ADT, from 19% with 1–6 months and 29% with 7–12 months to 33% with ≥13 months (*p* < 0.001). Unlike the other studies, the inclusion of only localized prostate malignancy cancer and rheumatoid arthritis as autoimmune manifestation could partly justify these conflicting results. 

In the growing context of cancer immunotherapy, the effects of ADT, particularly in earlier stages of disease, on the immune system and its impact on the success of emerging immunotherapies in prostate cancer [24] will require careful evaluation. In fact, in light of our preliminary evidence, immunotherapy for prostate cancer might be more efficient in HSPC, perhaps even more in association or in sequencing with ADT [25,26,27]. In addition, recent findings have shown the immunogenic effects of radiotherapy, including the increased infiltration of Tregs into the tumor microenvironment, leading to downregulation of the immune response [12,27,28]. Consequently, the combination of ADT and RT should be better explored in future studies of prostate cancer patients to better understand the emergence of autoimmune disorders and, especially, for potential clinical implications due to their common immune modulatory properties. In addition, immune-related biomarkers such as the systemic immune-inflammatory index (SII) and neutrophil-lymphocyte ratio (NLR) which already has shown a prognostic impact in CRPC treated with abiraterone or enzalutamide, should be correlated with the emergence of autoimmune disorders during these hormonal treatments [29,30].

In addition, with an exploratory purpose, we assessed the risk of developing second tumors in prostate cancer patients treated with ADT considering a second malignancy as the result of impaired immune regulation. We observed a higher incidence of second cancers, especially solid tumors, in prostate cancer patients treated with hormonal therapies mainly during HSPC status. Moreover, we might assume a potential immune modulation by external beam radiotherapy (as well a possible result by DNA damage from radiation), as demonstrated by the highest incidence of colorectal cancer and bladder cancer as second tumors in ours and other studies [31,32,33]—even urothelial carcinoma represents a well-known secondary effect of radiation therapy toxicity.

Several limitations in this study should be noted. First, we included a variety of different diseases such as autoimmune endocrine diseases, rheumatic diseases and systemic autoimmune diseases, characterized by heterogeneous mechanisms of action and different treatment approaches. Second, for almost all patients we have no appropriate information on the type of primary ADT, and it can be considered as a bias due to recent evidence [12,25] suggesting that the type of ADT can be a crucial factor in how immune responses change following androgen ablation. Additional limitations of this study are the lack of a control group of men of similar age who are not exposed to hormone therapy to assess the expected basal incidence of autoimmune disease for comparative purposes and the absence of similar comparative epidemiological studies in the metastatic setting. Moreover, this study could have a potential confounding factor related to abiraterone treatment in combination with a low dosage of prednisone, which is known to suppress inflammation and modulate immune responses. Lastly, this is a retrospective study and the number of autoimmune events is too small to draw definitive conclusions and final suggestions. However, two of the most important strengths of this study are large sample size and long-term follow-up, so further prospective studies are warranted to fully evaluate the relationship between prostate cancer and autoimmune diseases.

## 5. Conclusions

We first suggested that autoimmune comorbidities could have a prognostic role in prostate cancer patients treated with AR-directed therapies. This should be considered when deciding on therapeutic strategies in prostate cancer management, i.e., if patients have an autoimmune disease, should they be monitored more closely/receive different treatments to try to prolong the time to CRPC development. 

Finally, these findings hint towards the challenge to identify in future prospective larger studies the best approaches to combine or sequence ADT with other systemic treatments for prostate cancer.

## Figures and Tables

**Table 1:** Patient and treatment characteristics.

Characteristics	*n* = 844
Age, years	
median value (IQR)	70 (63–75)
Gleason score, *n* (%)	
6–7	297 (40.1)
≥8	444 (59.9)
Missing/Unknown	103
Visceral metastases, *n* (%)	
No	752 (89.1)
Yes	92 (10.9)
ECOG PS, *n* (%)	
0–1	788 (93.4)
2	56 (6.6)
Baseline PSA, ng/mL	
median value (IQR)	35.42 (8.64–115.40)
HSPC duration, months median value (IQR) ADT for HSPC, *n* (%)	29 (14–59) 844 (100)
CRPC duration, months median value (IQR) Prior therapeutic lines for CRPC, *n* (%)	22 (13–39)
1–2	764 (90.5)
>2	80 (9.5)
Potent AR-directed therapies for CRPC, *n* (%)	844 (100)
Abiraterone	477 (56.5)
Chemotherapy-naive	189 (39.6)
Post-docetaxel	288 (60.4)
Enzalutamide	367 (43.5)
Chemotherapy-naive	170 (46.3)
Post-docetaxel	197 (53.7)

ADT, androgen deprivation therapy; AR, androgen receptor; CRPC, castration resistant prostate cancer; ECOG, Eastern Cooperative Oncology Group; HSPC, hormone sensitive prostate cancer; IQR, interquartile range; N, number; PS, performance status; PSA, prostate-specific antigen.

**Table 2:** Baseline comorbidities of CRPC patients treated with abiraterone or enzalutamide.

Comorbidities	Total *n* (%)
No	277 (32.8)
Yes	567 (67.2)
Arterial hypertension	351 (41.6)
Metabolic syndrome	105 (12.4)
Dyslipidemia	99 (11.7)
Diabetes mellitus type 2	95 (11.3)
Ischemic cardiopathy	82 (9.7)
Arrhythmia	73 (8.6)
Vascular disease	38 (4.5)
Gastrointestinal disease	35 (4.1)
Neurological disease	35 (4.1)
Chronic obstructive pulmonary disease	28 (3.3)
Non-autoimmune thyropathy	19 (2.2)
Nephropathy	17 (2.0)
Autoimmune disease	36 (4.3)
Arthritis (8 rheumatoid, 5 psoriatic)	13 (1.5)
Autoimmune thyroiditis	12 (1.4)
Chronic inflammatory bowel disease (3 ulcerative colitis, 1 Crohn’s disease)	4 (0.5)
Psoriasis	4 (0.5)
Systemic vasculitis (2 giant cell arteritis, 1 vasculitis, 1 unspecified)	3 (0.4)
Second tumor (38 solid, 9 hematological)	47 (5.6)

**Table 3:** Multivariable analysis of predictors of PFS and OS.

	PFS		OS	
	HR (95% CI)	*p*	HR (95% CI)	*p*
Autoimmune disease (yes versus no)	1.37 (0.92–2.05)	0.119	1.63 (1.03–2.57)	0.037
Age (continuous variable)	1.002 (0.991–1.014)	0.690	1.015 (1.001–1.029)	0.042
Visceral metastasis (yes versus no)	1.27 (0.97–1.67)	0.085	1.82 (1.35–2.45)	<0.0001
ECOG PS (2 versus 0–1)	2.09 (1.53–2.86)	<0.0001	3.30 (2.37–4.58)	<0.0001
Pretreatment log PSA (continuous variable)	1.29 (1.23–1.36)	<0.0001	1.41 (1.32–1.49)	<0.0001
Previous chemotherapy (yes versus no)	1.87 (1.55–2.25)	<0.0001	1.74 (1.38–2.19)	<0.0001
Other comorbidities * (yes versus no)	1.04 (0.87–1.25)	0.645	1.05 (0.84–1.32)	0.638

* cardiovascular disease and/or metabolic syndrome. Abbreviations, CI, confidence interval; ECOG, Eastern Cooperative Oncology Group; HR, hazard ratio; OS, overall survival; PFS, progression-free survival; PS, performance status; PSA, prostate-specific antigen.

## Supplementary Materials

The following are available online at https://www.mdpi.com/2077-0383/9/6/1950/s1, Table S1: ICD-10 diagnostic codes of autoimmune diseases, Table S2: PSA response according to the presence of immune alterations, Table S3: univariate analysis of PFS and OS, Table S4: more common adverse events during treatment with abiraterone or enzalutamide.
