# Supplementary material for: Immune Modulation in Prostate Cancer Patients Treated with Androgen Receptor (AR)-Targeted Therapy
Source: J Clin Med. 2020 Jun 22;9(6):1950. doi: 10.3390/jcm9061950 (PMC7356925; doi:10.3390/jcm9061950)
Supplement: Supplementary file 1 [file jcm-09-01950-s001.pdf]

Supplementary Table 1. ICD-10 diagnostic codes of autoimmune diseases

**Autoimmune condition**	**Codes**
Systemic autoimmune diseases	Rheumatoid arthritis (ICD-10 M06.9), Systemic connective tissue disorders (ICD-10 M30-M36), Wegener's granulomatosis (ICD-10 M31.30, dermatomyositis (ICD-10 M33.90), systemic sclerosis (ICD-10 M34.0), Sjogren's syndrome (ICD-10 M35.0), polymyalgia rheumatic (ICD-10 M35.3), Raynaud's syndrome (ICD-10 I73.00), sarcoidosis (ICD-10 D86.9), autoimmune hemolytic anemia (ICD10 D59.0, D59.1), immune thrombocytopenic purpura (ICD-10 D69.2), Guillain–Barre´syndrome (ICD10 G61.0), myasthenia gravis (ICD10 G70.0), giant cell arteritis (ICD-10 M31-5), autoimmune disease, not elsewhere classified (ICD-10 D89.89)
Organ-specific autoimmune diseases	Hashimoto's thyroiditis (ICD-10 code E06.3), Grave's disease (ICD-10 code E05.0), ankylosing spondylitis (ICD-10 code M45.9), Celiac disease (ICD-10 code K90.0), inflammatory bowel disease (ICD-10 codes K50-K52), psoriasis and psoriatic arthritis (ICD-10 codes L40), autoimmune hepatitis (ICD10 K75.4), primary biliary cirrhosis (ICD10 K74.3), polyarteritis nodosa (ICD-10 M30.0), pemphigus vulgaris (ICD-10 codes L10.0), vasculitis limited to the skin, unspecified (ICD-10 L95.9)

Abbreviation: ICD-10, International Classification of Diseases-10th revision.

Supplementary Table 2. PSA response according to the presence of immune alterations

Abbreviations.n, number; PSA, prostate-specific antigen.

	**PSA Response Rate**			
	**No**	**Yes**	**Unknown/missing**	
	***n* (%)**	***n* (%)**	***n***	***p***
**Autoimmune disease**				
No	284 (94.0)	341 (96.0)	76	
Yes	18 (6.0)	14 (4.0)	4	0.232
**Risk of second tumour**				
No	284 (94.0)	341 (93.4)	76	
Yes	18 (6.0)	24 (6.6)	5	0.745

Supplementary Table 3. Univariate Analysis of PFS and OS

	PFS		OS	
	**HR (95% CI)**	***p***	**HR (95% CI)**	***p***
Autoimmune disease (yes *versus* no)	1.35 (0.91-2.01)	0.131	1.59 (1.03-2.47)	0.038
Age (continuous variable)	1.004 (0.993-1.014)	0.474	1.013 (1.000-1.026)	0.055
Visceral metastasis (yes *versus* no)	1.39 (1.07-1.79)	0.012	2.00 (1.51-2.65)	<0.0001
ECOG PS (2 *versus* 0-1)	2.69 (2.00-3.61)	<0.0001	4.13 (3.01-5.65)	<0.0001
Pretreatment log PSA (continuous variable)	1.33 (1.27-1.40)	<0.0001	1.42 (1.34-1.50)	<0.0001
Gleason score (≥8 vs <8)	1.14 (0.96-1.36)	0.135	1.12 (0.91-1.39)	0.278
Previous chemotherapy (yes *versus* no)	2.04 (1.72-2.42)	<0.0001	2.32 (1.87-2.88)	<0.0001

Abbreviations. CI, confidence interval; ECOG, Eastern Cooperative Oncology Group; HR, hazard ratio; OS, overall survival; PFS, progression-free survival; PS, performance status; PSA, prostate-specific antigen.

Supplementary Table 4. More common adverse events during treatment with abiraterone or enzalutamide

**Adverse Event (AE)**	**Total** *n* **(%)**
**No**	689 (81.6)
**Yes (any AE)**	155 (18.4)
Weakness	50 (5.9)
Nausea/vomiting	9 (1.0)
Diarrhea	7 (0.8)
Anorexia	8 (0.9)
Anemia/ thrombocytopenia	9 (1.0)
Uncontrolled arterial hypertension	15 (1.8)
Glucose intollerance	22 (2.6)
Metabolic syndrome	39 (4.6)
Heart failure	11 (1.3)
Fluid retention	10 (1.2)
Arthralgia/myalgia	15 (1.8)
Electrolyte abnormalities	4 (0.5)
Hypertransaminasemia	4 (0.5)
**AE Grade 3-4**	15 (1.8)
**Treatment discontinuation for AE**	9 (1.0)
**Life-threatening consequences/death**	2 (0.2)
